# Longitudinal multi-omics evidence reveals lung injury and concurrent disruption of intestinal flora and serum metabolism by cigarette smoke and influenza virus

**DOI:** 10.3389/fcimb.2026.1731390

**Published:** 2026-04-07

**Authors:** Zhihang Liu, Huameng Li, Yuting Xiang, Shaocong Ren, Wenchao Pan, Zihan Ling, Jianling Dong, Ziyao Liang, Jingyu Quan, Long Fan, Lin Lin, Lei Wu, Xuhua Yu

**Affiliations:** 1 The Second Affiliated Hospital of Guangzhou University of Chinese Medicine, State Key Laboratory of Traditional Chinese Medicine Syndrome/Department of Respiratory Disease, Guangzhou, Guangdong, China; 2 Department of Respiratory Disease, Zhuhai Hospital of Integrated Traditional Chinese and Western Medicine, Zhuhai, Guangdong, China

**Keywords:** acute exacerbation, chronic obstructive pulmonary disease, gut microbiota, gut-lung axis, influenza virus, metabolomics

## Abstract

**Background:**

Cigarette smoke (CS) exposure is the primary risk factor for chronic obstructive pulmonary disease (COPD), and respiratory viral infections, particularly influenza A virus (IAV), are major triggers of acute exacerbations of COPD (AECOPD). However, the dynamic interactions among pulmonary pathology, gut microbiota, and host metabolism during these episodes remains unclear. This study aimed to delineate the longitudinal characteristics of virus-induced AECOPD and identify potential biomarkers.

**Methods:**

Mice were exposed to cigarette smoke for eight weeks, followed by intranasal inoculation with IAV. A longitudinal assessment was conducted from day 1 to day 15 post-infection, integrating analyses of lung pathology, lung function, gut microbiome, and both serum and fecal metabolomes. Additionally, random forest modeling was employed to identify specific metabolic biomarkers associated with the acute exacerbation stage.

**Results:**

Mice exposed to cigarette smoke and IAV exhibited significant pulmonary immune cell recruitment, impaired lung function, and emphysematous changes, peaking at day 5 post-infection. By day 15, acute airway inflammation had subsided; however, interstitial immune cell infiltration, collagen deposition, and emphysema persisted. 16S rRNA sequencing revealed dynamic shifts in gut microbiota composition, with the abundance of *Intestinimonas* positively correlating with pulmonary inflammatory markers. Untargeted metabolomics demonstrated sustained downregulation of serum unsaturated fatty acid biosynthesis pathways from day 3 to day 15, and these metabolites were negatively correlated with lung inflammation. Random forest analysis identified 1-Methylnicotinamide (1-MNA) as a promising biomarker for distinguishing virus-triggered AECOPD, achieving an area under the curve (AUC) of 1.0.

**Conclusion:**

This study demonstrates that cigarette smoke combined with influenza infection induces persistent lung injury alongside concurrent disruption of intestinal flora and serum metabolism. The findings show that gut microbiota and metabolites are potential biomarkers and supplementation with unsaturated fatty acids may represent a novel therapeutic strategy for virus-induced AECOPD.

## 1 Introduction

Cigarette smoke exposure is the primary risk factor for chronic obstructive pulmonary disease (COPD), driving persistent airway inflammation and progressive emphysema (Morse and Rosas, 2014; GBD Chronic Respiratory Disease Collaborators, 2020). Respiratory viral infections are among the most common triggers of acute exacerbations of COPD (AECOPD) (Stolz et al., 2019; Konstantinoudis et al., 2022; Li et al., 2022). The worldwide mean prevalence of respiratory tract viral infections was 34.1% in AECOPD (Mohan et al., 2010). Among these infections, the influenza virus is the most frequently detected virus in Asia (Mohan et al., 2010). Virus-positive episodes are associated with more severe symptoms, prolonged recovery, and higher hospitalization rates (Seemungal et al., 2001; White et al., 2003; Singanayagam et al., 2012; George et al., 2014). Although most respiratory viral infections are self-limiting, the inflammation and pathological changes induced by viral infection indicate that respiratory viral infections in COPD warrant greater attention. However, the pathological characteristics of AECOPD caused by the influenza virus remain unclear, and the indications for antiviral drugs in the treatment of virus-induced AECOPD have not been definitively established (Cai et al., 2014).

Metabolomic profiling and gut microbiota analyses present promising approaches to address this knowledge gap. Alterations in metabolites and the gut microbiota have been extensively utilized to characterize disease onset and progression. Since the introduction of the “gut–lung axis”, bidirectional communication between intestinal microbes and the pulmonary system has been extensively documented (Budden et al., 2017; Ma et al., 2022). Numerous clinical and experimental studies demonstrate that COPD patients exhibit significant gut dysbiosis, and disrupted microbial metabolites actively contribute to disease pathophysiology (Qu et al., 2022; Wang L. et al., 2023). Recent clinical investigations further reveal a rapid restructuring of the gut microbiota during AECOPD episodes (Wu et al., 2021; Yan et al., 2024). Nevertheless, how the intestinal microbiota, host metabolism, and pulmonary inflammation dynamically interact during virus-induced AECOPD remain largely unexplored. Therefore, a multi-time-point, systems-level dynamic mapping of concurrent changes in gut microbiota composition, serum metabolome, and pulmonary inflammation is essential. Only by continuously tracking these evolving alterations can the pathological trajectory of virus-induced AECOPD be accurately delineated, and reliable biomarkers as well as novel therapeutic targets be identified.

Because population-level omics analyses are easily confounded by diet, sex, and lifestyle factors, standardized animal models remain the primary method (Buchwald et al., 2017; Tsonaka et al., 2020; Wen et al., 2020; Zhuge et al., 2022; Chen et al., 2023). Mice were exposed to cigarette smoke for eight weeks and subsequently intranasally inoculated with influenza A virus (IAV). We then longitudinally assessed pulmonary inflammation, emphysema, lung function, gut microbiome, and metabolic profiles from day 1 to day 15 post-infection. Finally, random forest modeling was employed to identify specific metabolic characteristics associated with virus-triggered AECOPD.

## 2 Methods

### 2.1 Animals

Specific pathogen-free (SPF) female BALB/c mice, aged 6 to 8 weeks and weighing 18–20 g, were purchased from Beijing Vital River Laboratory Animal Technology Co., Ltd. The animals were housed under SPF conditions at the Experimental Animal Center of Guangdong Huawei Testing Co., Ltd. All experiments were conducted in accordance with the guidelines of the National Health and Medical Research Council of China and were approved by the Guangdong Provincial Hospital of Traditional Chinese Medicine Laboratory Animal Ethics Committee (IACUC number: 2021066).

### 2.2 Cigarette smoke exposure

**Figure 1:**
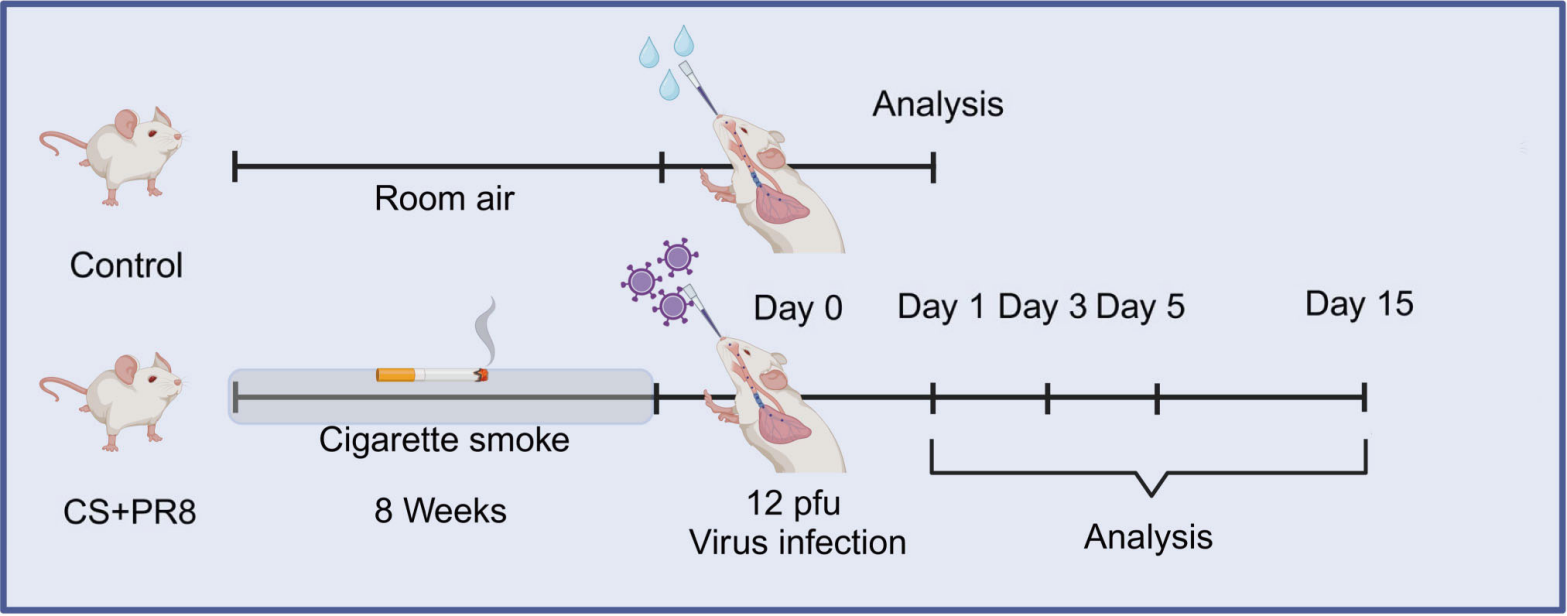
Experimental design for the AECOPD mouse model induced by long-term cigarette smoke exposure combined with IAV infection.

The experimental flowchart is presented in **Figure 1**. Mice were exposed to smoke generated by burning filter-tipped DaQianMen cigarettes (Shanghai Tobacco Group Co., Ltd., Shanghai, China) within an 18-L plastic chamber housed inside a Class II biosafety cabinet, as previously described (Yu et al., 2019). Cigarette smoke (CS) was generated with a 60-ml tidal volume over 10 seconds to simulate human smoking inhalation volume and the typical burn rate of a cigarette. Mice were exposed to smoke from six cigarettes per day for 8 weeks, following a protocol in which they inhaled CS for 1 hour twice daily, at 10 a.m. and 3 p.m. The smoke generated in the chamber had a total suspended particulate mass concentration of 540 mg·m^−3^. Sham-exposed mice were placed in an identical 18-L plastic chamber but were not exposed to CS. The commercially available filter-tipped DaQianMen cigarettes used contained the following: 11 mg or less of tar, 0.8 mg or less of nicotine, and 13 mg or less of carbon monoxide.

### 2.3 IAV infection

After eight weeks of exposure to CS or sham treatment, mice were anesthetized with isoflurane on day 0 and administered 12 plaque-forming units (PFU) of A/PR/8/34 (H1N1) influenza virus via nasal aspiration in 30 μl of minimal essential Eagle’s medium (MEM), as previously described (Gualano et al., 2008; Ding et al., 2017; Yu et al., 2019). An equal volume of MEM was administered intranasally to vehicle-control mice. The mice were then sacrificed on days 1, 3, 5, and 15 after viral infection, respectively.

### 2.4 Lung function

Before the mice were sacrificed, whole-body plethysmography (EMMS, Bordon, Hants) was used to assess lung function while the mice were awake. The parameters measured included tidal volume (TV), expiratory time (TE), enhanced pause (Penh), relaxation time (Tr), expiratory flow at 50% of tidal volume (EF50), and peak expiratory flow (PEF). Each mouse was placed individually into one of four chambers and allowed to acclimate for five minutes. Measurements were recorded once per second for five minutes.

### 2.5 Bronchoalveolar lavage fluid and lung collection

After euthanasia using sodium pentobarbital, the primary trachea and lungs of each mouse were cannulated and flushed once with 400 µl of phosphate-buffered saline (PBS), followed by three additional flushes with 300 µl of PBS each. Total and differential leukocyte counts were obtained from the resulting bronchoalveolar lavage fluid (BALF). Cell types were identified based on standard morphological criteria. Blood was removed from the lungs by perfusing 5 ml of PBS through the right ventricle of the heart. The lungs were then rapidly excised en bloc, rinsed in PBS, blotted, snap-frozen in liquid nitrogen, and stored at −80 °C until further analysis.

### 2.6 Hematoxylin and eosin staining, and Masson’s trichrome staining

After the BALF was taken and the blood removed, the upper lobe of the left lung from each mouse was extracted and preserved for at least 24 hours at room temperature in 4% paraformaldehyde. Following fixation, the lung tissues were embedded in paraffin blocks, sectioned into 4-micrometer-thick slices, treated with xylene to remove the paraffin, and then rehydrated using ethanol. Subsequently, the tissue samples were rinsed with distilled water for five minutes. They were then stained with haematoxylin and eosin (Servicebio, Wuhan, China) to assess inflammatory cell infiltration or stained with Masson’s trichrome to evaluate fibrosis formation. The stained sections were examined and photographed using an automated imaging microscope (ECLIPSE Ti2-E, Nikon, Tokyo, Japan).

For hematoxylin and eosin (H&E) staining, the final inflammation score for each lung was determined by averaging the scores from nine distinct regions of the lung image. The scoring criteria were based on Curtis’s study (Curtis et al., 1991), with detailed criteria provided in **Supplementary Table 1**. This scoring method was previously described in our previous study (Yu et al., 2021). Briefly, lung tissue sections were divided into nine equal regions. Each region was independently scored for alveolar, airway, and vascular inflammation, and the final score for each category was calculated as the average of the nine regional scores.

For Masson’s trichrome staining, the region of interest (ROI) was defined within the hilar region, centered on the lobar or segmental bronchus and its accompanying pulmonary vessels. The ROI was set as a rectangle measuring 1500 μm in length and 900 μm in width. Images were processed using ImageJ/Fiji. Collagen fibers were segmented by color thresholding in the HSB color space to select blue-green areas corresponding to collagen. The collagen area fraction was calculated as the ratio of the collagen area to the total ROI area and expressed as a percentage.

### 2.7 Histological assessment of emphysema

The criteria were based on Steven et al (Fung et al., 2024). Five randomly selected fields at 200× magnification in the lung regions—avoiding the hilum, vasculature, and airways—were analyzed. A single 10 × 10 square grid, with each small square measuring 100 μm × 100 μm, was created and overlaid on an area within each field. The number of alveolar walls intersecting each horizontal grid line was then counted. The mean linear intercept (Lm) was calculated by first subtracting the distance occupied by blood vessels and airways on each horizontal line from the total length of all horizontal grid lines, then dividing the remaining distance by the total number of alveolar surface intersections counted. The average Lm across all five grids was used as the final Lm for each lung sample.

### 2.8 Immunofluorescence staining

Lung tissue sections (4 μm) were deparaffinized and rehydrated, followed by heat-induced antigen retrieval. Endogenous peroxidase activity was blocked with 3% hydrogen peroxide in methanol. A tyramide signal amplification (TSA) multiplex immunofluorescence staining technique was employed to simultaneously visualize CD3, CD68, and MPO. The sections were sequentially incubated with primary antibodies at 4 °C overnight. The primary antibodies used were rabbit anti-CD3 (1:2500; Servicebio, Wuhan, China), rabbit anti-CD68 (1:8000; Servicebio, Wuhan, China), and rabbit anti-MPO (1:2500; Servicebio). Following each primary antibody incubation, a horseradish peroxidase (HRP)-conjugated goat anti-rabbit polymer antibody (S-vision; Servicebio) was applied, followed by incubation with the corresponding fluorescent tyramide substrates. Specifically, iF555-Tyramide, iF488-Tyramide, and iF647-Tyramide (Servicebio) were used for CD3, CD68, and MPO labeling, respectively. Between each staining round, an antibody elution buffer (Servicebio) was used to remove antibody complexes while preserving the covalently bound fluorescent signals. Nuclei were counterstained with DAPI (Servicebio). Finally, sections were mounted with an anti-fluorescence quenching mounting medium and scanned using a digital slide scanner (Pannoramic MIDI, 3DHISTECH). Quantitative analysis was performed by randomly selecting five fields of view (400× magnification) within the lung parenchyma, avoiding the hilum, vascular system, and airways. An 800 μm × 450 μm rectangle was created in each field, and the number of positive cells within the rectangle was counted. The average value of the five rectangles was used as the final statistical result for each lung sample.

### 2.9 RNA extraction and quantitative real-time PCR

FastPure Complex Tissue/Cell Total RNA Isolation Kit (Vazyme, Nanjing, China) was used to extract total RNA from mouse lung tissues, which was quantified using a NanoDrop™ 2000 Spectrophotometer (Thermo Fisher Scientific, Waltham, MA, USA). The RNA was subsequently reverse transcribed into complementary DNA (cDNA) using the Evo M-MLV RT Premix for qPCR (Accurate Biotechnology (Hunan) Co., Ltd., China). Quantitative RT–PCR was performed with the SYBR Green Premix Pro Taq HS qPCR Kit (Rox Plus) (Accurate Biotechnology (Hunan) Co., Ltd., China) on an ABI Q7 RT–PCR System (Applied Biosystems, Foster City, CA, USA). GAPDH mRNA was used as an internal control. The threshold cycle (Ct) value represents the PCR cycle number (out of 40) at which the measured fluorescent signal exceeds a calculated background threshold, indicating amplification of the target sequence. The Ct value is inversely proportional to the number of input target copies present in the sample. Ct values were analyzed using the relative quantification method and expressed relative to GAPDH mRNA levels. Primer sequences are listed in **Supplementary Table 2**.

### 2.10 16S rRNA gene sequencing

Cecal stool DNA was extracted using the ALFA-SEQ Advanced Stool DNA Kit and quantified with a NanoDrop One and a Qubit 4.0. The V4 hypervariable region of the 16S rDNA was amplified using primers 515F/806R, purified, and assessed on a Qsep400 system. Amplicons were sequenced (PE250) on Illumina or MGI platforms. Raw reads were initially quality-filtered and trimmed with fastp (-W 4 -M 20), followed by primer removal using cutadapt. Paired-end reads were merged with usearch (-fastq_mergepairs, ≥16 bp overlap, ≤5 mismatches), re-filtered with fastp. Finally, sequences were clustered into operational taxonomic units (OTUs) using UPARSE (Edgar, 2013).

### 2.11 Serum and stool metabolite extraction and preparation

Serum and stool samples were transferred to EP tubes. After adding 200 μL of extraction solution (acetonitrile:methanol, 1:1, v/v) containing an isotopically labeled internal standard mixture, the samples were vortexed for 30 s, sonicated for 10 min in an ice-water bath, and incubated for 1 h at −40 °C to precipitate proteins. The isotopically labeled internal standards and their final concentrations were as follows: N-(carboxymethyl)-N,N,N-trimethyl-d_9_-ammonium chloride (CAS 285979-85-3, 0.4 μmol/L), succinic-2,2,3,3-d_4_ acid (CAS 14493-42-6, 3.0 μmol/L), N-benzoyl-d_5_-glycine (CAS 53518-98-2, 0.6 μmol/L), 4-aminobutyric-2,2,3,3,4,4-d_6_ acid (CAS 70607-85-1, 2.0 μmol/L), and nicotinamide-2,4,5,6-d_4_ (CAS 317841-88-7, 0.6 μmol/L). The samples were then centrifuged at 12,000 rpm (RCF = 13,800 × g, radius = 8.6 cm) for 15 min at 4 °C. The resulting supernatant was transferred to fresh glass vials for LC-MS analysis. The quality control (QC) sample was prepared by pooling equal aliquots of supernatants from all study samples and was injected at regular intervals throughout the analytical batch (one QC after every four study samples) to monitor system stability.

### 2.12 LC-MS/MS analysis for serum and stool metabolites

The supernatant was then analysed by liquid chromatography-tandem mass spectrometry (LC-MS/MS) to determine metabolites in the serum. LC-MS/MS analyses were performed using an UHPLC system (Vanquish, Thermo Fisher Scientific) equipped with a Waters ACQUITY UPLC BEH Amide column (2.1 mm × 50 mm, 1.7 μm), coupled to an Orbitrap Exploris 120 mass spectrometer (Orbitrap MS, Thermo). The mobile phase consisted of 25 mmol/L ammonium acetate and 25 mmol/L ammonium hydroxide in water (pH 9.75) (A) and acetonitrile (B). Chromatographic separation was achieved using the following gradient program at a flow rate of 0.50 mL/min: 0–0.25 min, 95% B; 0.25–3.50 min, 95% to 65% B; 3.50–4.00 min, 65% to 40% B; 4.00–4.50 min, 40% B; 4.50–4.55 min, 40% to 95% B; 4.55–6.00 min, 95% B for column re-equilibration. The autosampler temperature was maintained at 4 °C, and the injection volume was 2 μL. The Orbitrap Exploris 120 mass spectrometer was utilized for its capability to acquire MS/MS spectra in information-dependent acquisition (IDA) mode, controlled by the acquisition software (Xcalibur, Thermo). In this mode, the software continuously evaluates the full-scan MS spectrum. The ESI source conditions were set as follows: sheath gas flow rate, 50 Arb; auxiliary gas flow rate, 15 Arb; capillary temperature, 320 °C; full MS resolution, 60,000; MS/MS resolution, 15,000; collision energy, SNCE 20/30/40; spray voltage, 3.8 kV (positive mode) or −3.4 kV (negative mode), respectively.

### 2.13 Statistical analysis

The data obtained from all experiments were subjected to one-way analysis of variance (ANOVA) followed by Tukey’s multiple comparison test. Results are presented as means ± standard error of the mean (SEM). Correlation analysis was performed using Spearman’s rank correlation. Statistical analyses were conducted using GraphPad Prism version 10.0 for Windows (GraphPad, San Diego, CA, United States), which was also used to generate the graphs. P values less than 0.05 were considered statistically significant.

## 3 Results

### 3.1 Cigarette smoke combined with IAV infection induces the recruitment of immune cells in the lungs

**Figure 2:**
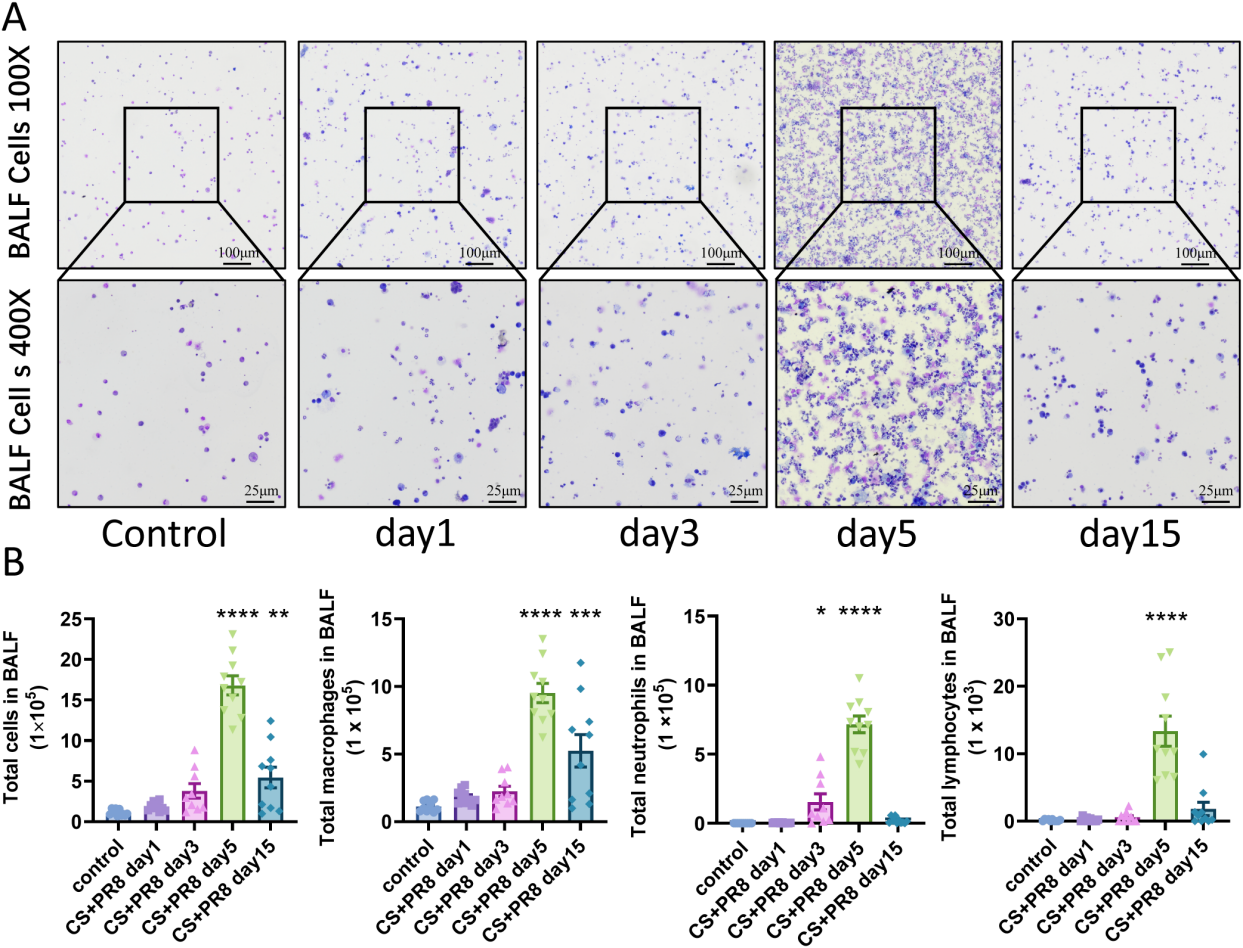
Increased inflammatory response in the airways following IAV infection in cigarette smoke-exposed mice. **(A)** Representative images of Kwik-Diff-stained cytospin preparations from bronchoalveolar lavage fluid (BALF) of mice in each group. **(B)** Total cell counts and numbers of macrophages, neutrophils, and lymphocytes in BALF. Data are presented as mean ± standard error. One-way analysis of variance combined with Tukey’s multiple comparison test was used to assess statistical significance. Compared with the control group: *p < 0.05, **p < 0.01, ***p < 0.001, ****p < 0.0001; n = 9–10.

Mice were exposed to either CS or ambient room air for 8 weeks and subsequently infected intranasally with IAV. The immune cells recruited to the airway and alveolar cavity were examined (**Figure 2A**). The number of inflammatory cells in the BALF peaks on day 5 post-infection, with significant increases in macrophages, neutrophils, and lymphocytes (**Figure 2B**). Additionally, a sustained high level of macrophage aggregation was observed in the BALF on day 15 after infection.

**Figure 3:**
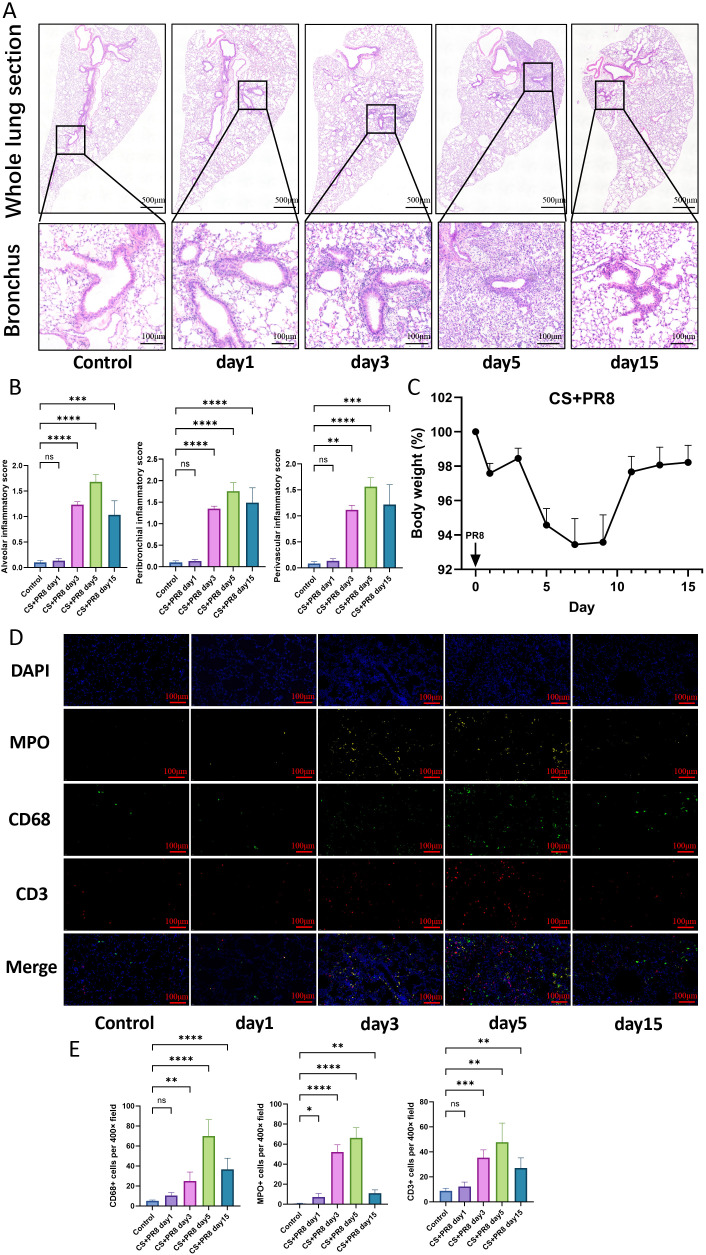
Increased inflammatory response in pulmonary tissues following IAV infection in cigarette smoke-exposed mice. **(A)** Representative images of hematoxylin and eosin (H&E) staining of whole lung sections. **(B)** Pneumonia scores in the alveolar, peribronchial, and perivascular regions. **(C)** Body weight changes normalized to body weight before PR8 instillation. **(D)** Representative immunofluorescence images of lung tissue (×400). **(E)** Quantification of CD68^+^ (macrophages), MPO^+^ (neutrophils), and CD3^+^ (lymphocytes) positive signals. Data are presented as mean ± standard error. One-way analysis of variance combined with Tukey’s multiple comparison test was used to assess statistical significance. Compared with the control group, *p < 0.05, **p < 0.01, ***p < 0.001, ****p < 0.0001.

Immune cells remaining in the lung interstitium were also observed through HE staining and immunofluorescence staining. The results demonstrated a significant increase in infiltrating inflammatory cells around the airways, blood vessels, and alveoli—from the 3rd to the 15th day after IAV infection—particularly around the pulmonary hilum (**Figures 3A, B, D, E**).

Additionally, there was a significant reduction in body weight, as shown in **Figure 3C**. Throughout the course of the viral infection, the mice lost approximately 10% of their baseline body weight, and this weight loss had not fully recovered even 15 days post-infection.

### 3.2 Cigarette smoke combined with IAV infection induces inflammation and immune responses in lung tissue

We subsequently evaluated the mRNA expression levels of pulmonary inflammatory cytokines, chemokines, and interferons (**Table 1**). In the CS+IAV mice, mRNA expression levels of cytokines (IL-6, TNF-α, IL-1β), pro-inflammatory chemokines (CCL2, CXCL1, CXCL2), interferons (IFN-α, IFN-β, IFN-γ, IFN-λ), and interferon-stimulated genes (MX2, OAS1, CXCL10), as well as viral nucleoprotein (NP), were increased on day 3 post-infection (**Table 1**). Most of these indicators significantly decreased by day 5 post-infection compared to day 3, except for IFN-α, IFN-β, and IFN-λ. By day 15 post-infection, the mRNA expression levels of these markers were not significantly different from those in the control group.

**Table 1:** CS exposure and IAV infection enhance cytokine expression in the lungs.

Gene	Control	CS+PR8 day1	CS+PR8 day3	CS+PR8 day5	CS+PR8 day15
TNF-α	1.09 ± 0.40	1.60 ± 0.58	9.03 ± 5.43^****^	5.31 ± 1.91^**^	2.68 ± 0.69
IL-1β	1.10 ± 0.46	1.06 ± 0.74	7.39 ± 5.29^****^	2.08 ± 1.06	1.50 ± 0.70
IL-6	1.10 ± 0.44	1.42 ± 0.41	105.59 ± 75.71^****^	46.83 ± 28.49^*^	2.97 ± 0.93
CCL2	1.11 ± 0.53	1.97 ± 0.72	221.24 ± 163.15^****^	99.16 ± 57.27^*^	4.35 ± 1.63
CXCL1	1.13 ± 0.58	3.62 ± 2.37	46.62 ± 32.74^****^	25.00 ± 17.15^*^	3.29 ± 1.65
CXCL2	1.12 ± 0.51	3.83 ± 1.48	44.06 ± 39.50^***^	14.02 ± 7.89	2.99 ± 1.48
CXCL10	1.06 ± 0.33	3.01 ± 3.61	785.15 ± 595.31^****^	201.57 ± 111.04	12.82 ± 11.03
IFN-α	2.11 ± 1.99	2.58 ± 1.95	8.08 ± 4.87^**^	12.47 ± 2.88^****^	4.36 ± 3.60
IFN-β	1.95 ± 1.88	2.23 ± 1.70	43.96 ± 30.51^****^	36.43 ± 17.42^****^	3.26 ± 2.57
IFN-γ	1.19 ± 0.73	1.23 ± 0.30	55.80 ± 49.47^****^	7.79 ± 4.23	6.33 ± 3.69
IFN-λ	2.16 ± 1.85	4.02 ± 1.61	10.35 ± 5.66^****^	8.93 ± 2.07^***^	3.23 ± 2.36
H1N1NP	3.54 ± 5.42	57.16 ± 94.90	12200 ± 10968^**^	11754 ± 9490^**^	18.19 ± 23.70
MX2	1.13 ± 0.62	1.18 ± 0.22	37.14 ± 19.19^****^	8.50 ± 3.50	1.42 ± 0.55
OAS1	1.04 ± 0.29	1.21 ± 0.30	7.93 ± 3.65^****^	1.84 ± 0.84	1.16 ± 0.33
MMP-12	1.29 ± 0.90	1.66 ± 0.68	2.13 ± 1.44	4.28 ± 0.68^****^	2.12 ± 1.19

The mRNA expression levels of cytokines (IL-6, TNF-α, IL-1β), pro-inflammatory chemokines (CCL2, CXCL1, CXCL2, and CXCL10), interferons (IFN-α, IFN-β, IFN-γ, and IFN-λ), viral nucleoprotein (NP), and interferon-stimulated genes (MX2, OAS1) were measured in lung tissues. Data are presented as mean ± standard deviation. One-way analysis of variance followed by Tukey’s multiple comparison test was used to assess statistical significance. Compared with the control group, *p < 0.05, **p < 0.01, ***p < 0.001, ****p < 0.0001; n = 9–10.

### 3.3 Cigarette smoke combined with IAV infection induces changes in lung structure

**Figure 4:**
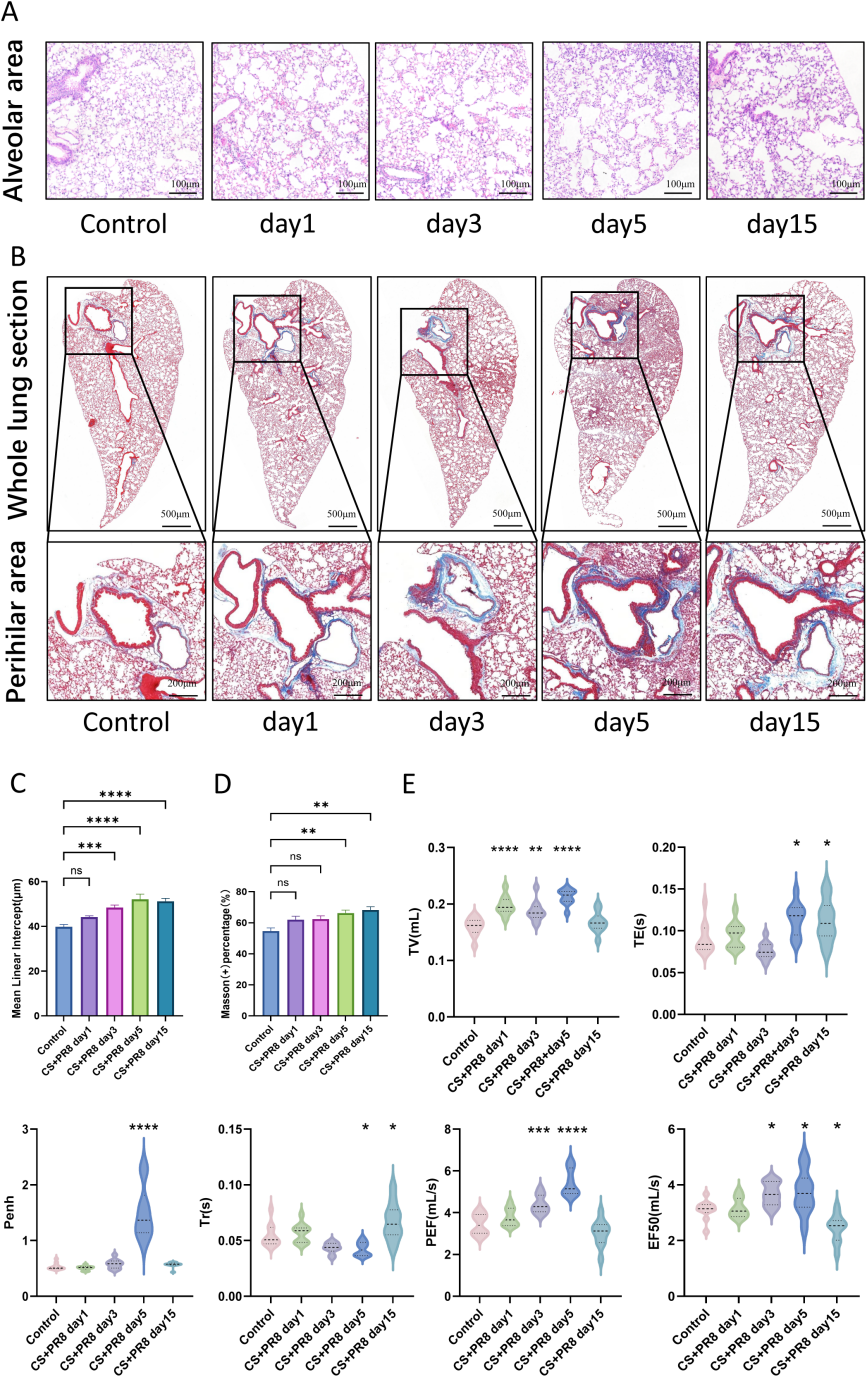
CS exposure and IAV infection induce changes in lung structure and function. **(A)** Representative images of Hematoxylin and eosin (H&E) staining of the alveolar area. **(B)** Representative images of Masson’s trichrome staining of whole lungs and hilum of the lungs. **(C)** Mean linear intercept (Lm) values (n = 10). **(D)** Quantification of peribronchial collagen deposition using Masson’s trichrome staining score (n = 3). **(E)** Pulmonary dysfunction in mice exposed to long-term cigarette smoke combined with influenza virus infection. Tidal volume (TV), expiratory time (TE), enhanced pause (Penh), relaxation time (Tr), peak expiratory flow (PEF), and mid-expiratory flow rate (EF50) were measured (n = 10). Data are presented as mean ± standard error. One-way analysis of variance followed by Tukey’s multiple comparison test was used to assess statistical significance. Compared with the control group, *p < 0.05, **p < 0.01, ***p < 0.001, ****p < 0.0001.

To evaluate whether an 8-week exposure to CS and IAV infection induced emphysema, the mean linear intercept (Lm) was measured on H&E-stained whole lung sections to quantify airspace enlargement (**Figure 4A**). Lung sections from mice not exposed to CS exhibited well-structured and intact alveoli, whereas CS exposure combined with IAV infection caused extensive disruption of alveolar septa five days post-infection, consistent with the significant increase in MMP-12 observed at this time point (**Table 1**). This disruption was even more pronounced fifteen days after infection (**Figures 4A, C**).

In contrast, virus-challenged, CS-exposed mice exhibited a pronounced increase in blue-stained collagen around the hilum on day 5 post-infection. Notably, although airway inflammation had subsided by day 15, collagen deposition continued to increase (**Figures 4B, D**).

### 3.4 Cigarette smoke combined with IAV infection induces changes in lung function

At five days post-infection, a significant increase in tidal volume (TV), enhanced pause (Penh), peak expiratory flow (PEF), and expiratory flow at 50% tidal volume (EF50) was observed, indicating increased airway resistance and obstructive ventilatory disorder in the mice (**Figure 4E**). Furthermore, at 15 days post-infection, expiratory time (TE) and relaxation time (Tr) remained significantly longer than those of uninfected mice, suggesting the possibility of persistent lung function impairment (**Figure 4E**).

### 3.5 Cigarette smoke combined with IAV infection alters the composition of the gut microbiota

**Figure 5:**
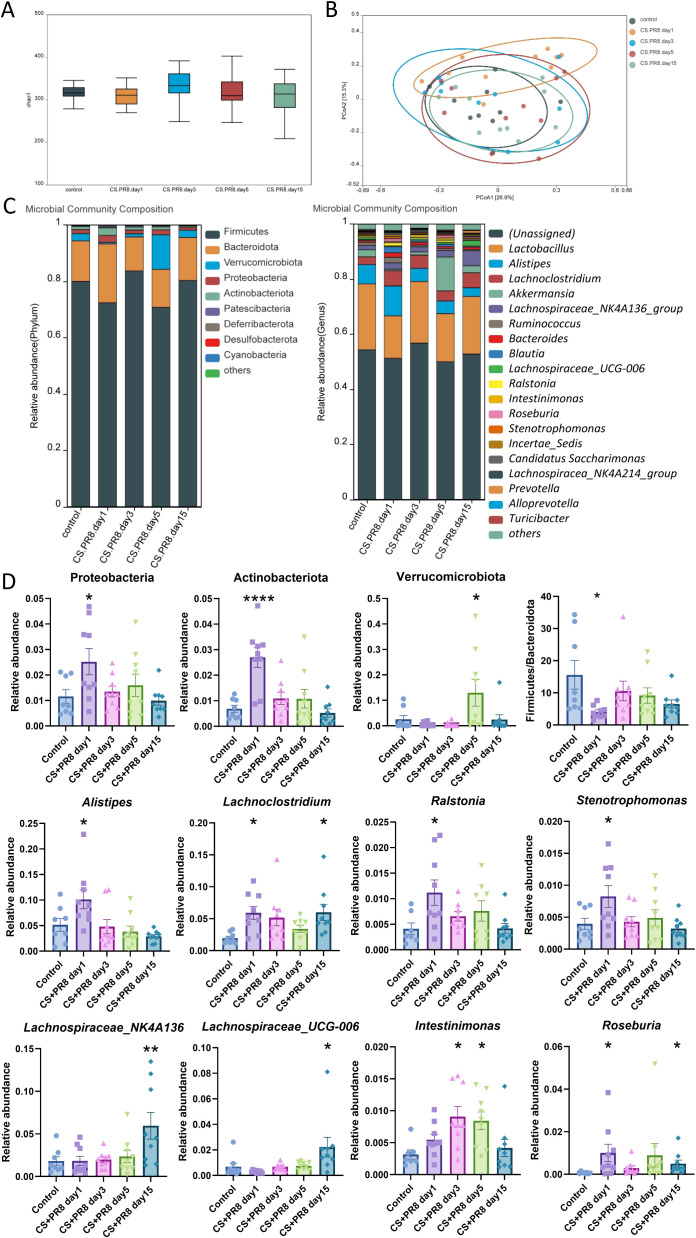
Altered gut microbiota composition following IAV infection in CS-exposed mice. **(A)** Shannon index of gut microbiota (α-diversity). **(B)** Principal Coordinates Analysis (PCoA) of gut microbiota (β-diversity). **(C)** The top 10 phyla and top 20 genera of gut microbiota are color-coded, highlighting key genera. **(D)** Relative abundance of gut microbiota at the phylum and genus levels. Data are presented as mean ± standard error. One-way analysis of variance combined with Tukey’s multiple comparison test was used to assess statistical significance. Compared with the control group: *p < 0.05, **p < 0.01, ***p < 0.001, ****p < 0.0001; n = 8–9.

Multiple studies have demonstrated that exposure to CS or IAV infection modifies the composition of the gut microbiota in mice (Yildiz et al., 2018; Sencio et al., 2020; Lai et al., 2022; Budden et al., 2024). However, the combined impact of cigarette smoke and IAV infection on the intestinal flora of mice remains unclear. To monitor the dynamics of gut microorganisms, we employed 16S rRNA gene sequencing to compare changes in the gut microbiota. Forty-four cecal content samples were collected from control and model mice on days 1, 3, 5, and 15, respectively. A total of 9,021 OTUs were clustered (**Supplementary Figure 1A**). The sample size and sequencing data available then were adequate, as indicated by the rarefaction curve (**Supplementary Figure 1B**). In the present study, we observed no significant differences in the Chao1 indexes between the CS-IAV mice and control mice (**Figure 5A**). Additionally, β-diversity principal coordinate analysis (PCoA) based on Bray-Curtis dissimilarity and Euclidean distance revealed a gradual recovery trend after day 3 (**Figure 5B**).

Subsequently, we assessed the relative abundance of microbial species at several stages to identify characteristic microbes following IAV infection. At the phylum level, the proportions of Proteobacteria and Actinobacteriota were increased on the first day post-infection compared to the control group, while the proportions of Firmicutes, Bacteroidetes, and Firmicutes/Bacteroidetes ratio were decreased on the same day. By day 5 post-infection, Verrucomicrobiota was markedly enriched (**Figures 5C, D**).

At the genus level, *Alistipes*, *Lachnoclostridium*, *Ralstonia*, *Roseburia*, and *Stenotrophomonas* showed increased abundance on the first day post-infection compared to the control group (**Figures 5C, D**). The abundance of *Intestinimonas* was enriched on days 3 and 5 post-infection. Similarly, the relative abundance of *Akkermansia* increased on day 5. *Lachnoclostridium*, *Lachnospiraceae_NK4A136*, *Lachnospiraceae_UCG-006* and *Roseburia* exhibited significant growth on day 15 post-infection.

**Figure 6:**
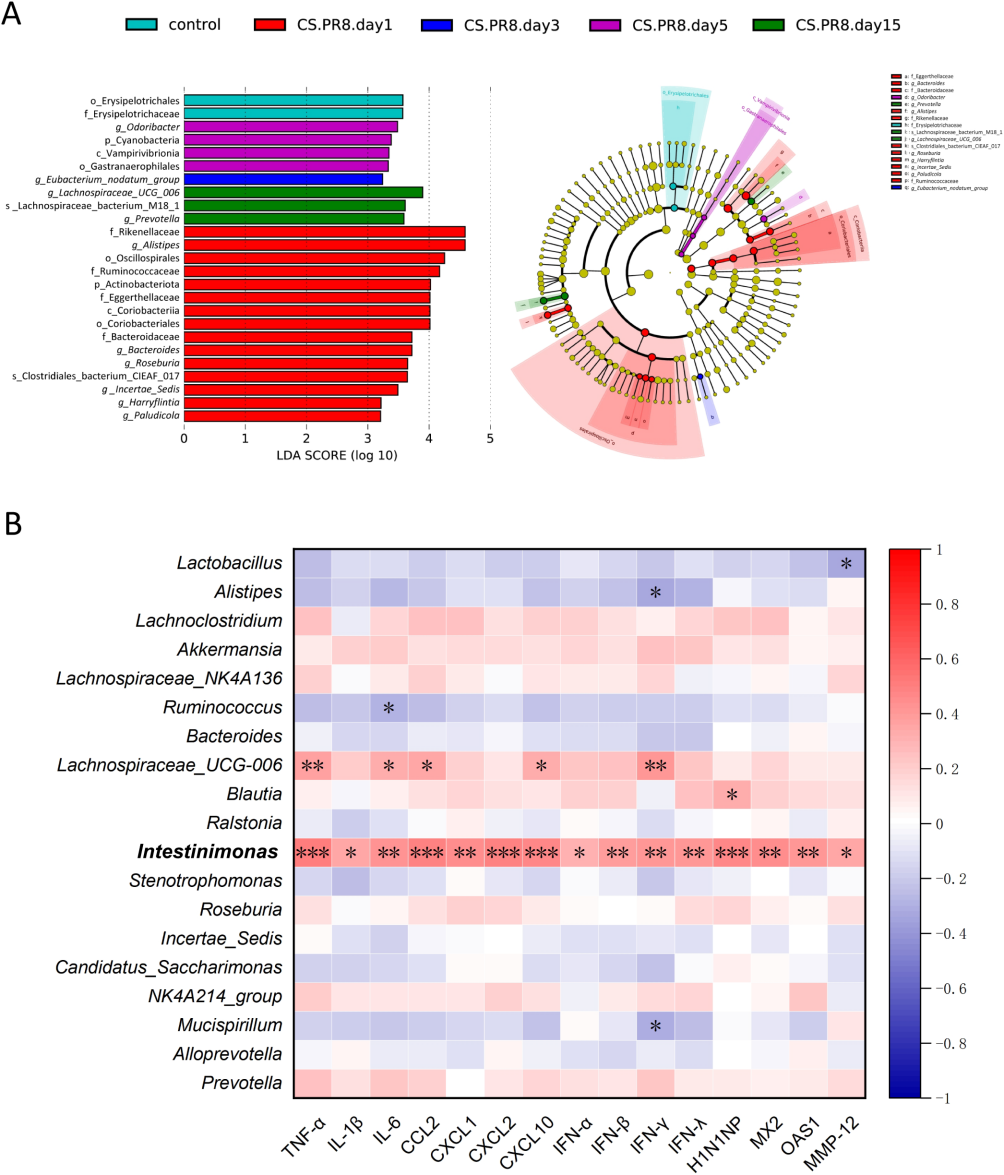
Linear discriminant analysis effect size (LEfSe) of gut microbiota. **(A)** LEfSe score plot of gut microbiota and LDA score plot of gut microbiota. **(B)** Heatmap showing the correlation between gut microbiota at the genus level and pulmonary cytokines. *P < 0.05, **P < 0.01, ***P < 0.001, ****P < 0.0001.

A linear discriminant analysis of effect size (LEfSe) was conducted to identify the microorganisms present at each time point following the onset of AECOPD. The results are illustrated by the LDA score plot and branching diagram (**Figure 6A**) with an LDA fold = 3.2 and P <0.05. In the early stages of infection, f_Rikenellaceae, *g_Alistipes*, and o_Oscillospirales, among others exhibited a considerable increase. Fifteen days post-infection, *g_Lachnospiraceae_UCG_006*, s_Lachnospiraceae_bacterium_M18_1, and *g_Prevotella* exhibited considerable enrichment.

To investigate the relationship between key gut microbiota and lung inflammation, we performed a correlation analysis on the top 20 dominant genera based on their relative abundance in the intestine and the results of quantitative real-time PCR. *Intestinimonas* were positively correlated with most inflammatory markers in the lungs following IAV infection in CS-exposed mice (**Figure 6B**).

### 3.6 Cigarette smoke combined with IAV infection altered serum and fecal metabolomic signatures

**Figure 7:**
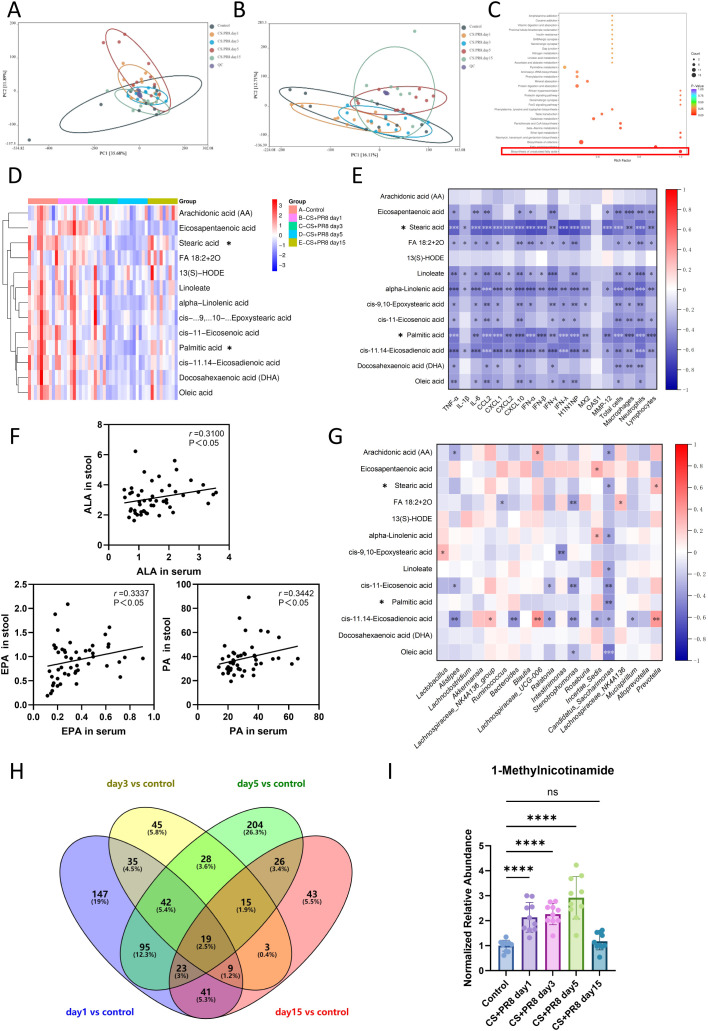
Altered serum and fecal metabolomic signatures following IAV infection in CS-exposed mice. **(A)** Principal Component Analysis (PCA) of serum metabolites in mice. **(B)** Principal Component Analysis (PCA) of fecal metabolites in mice. **(C)** KEGG enrichment analysis of differential serum metabolites on day 5 post-infection. **(D)** Heatmap of differential expression of metabolites in the serum unsaturated fatty acid pathway between control and CS-exposed mice. **(E)** Heatmap showing the correlation between serum metabolites in the unsaturated fatty acid metabolism pathway and pulmonary cytokines. **(F)** Correlation of metabolites in the biosynthesis of unsaturated fatty acid metabolic pathway between feces and serum (positive correlations only). **(G)** Heatmap showing the correlation between gut microbiota at the genus level and fecal metabolites in the biosynthesis of the unsaturated fatty acid pathway. **(H)** Venn diagram of differential serum metabolites in CS-exposed mice. **(I)** Normalized relative abundance variation of 1-Methylnicotinamide in serum. *Saturated fatty acids, such as palmitic and stearic acid, serve as precursor metabolites in the biosynthesis of unsaturated fatty acids, being converted into palmitoleic and oleic acid, respectively. *P < 0.05, **P < 0.01, ***P < 0.001, ****P < 0.0001.

Non-targeted metabolomics was used to investigate the metabolic characteristics of serum and feces in mice. **Figure 7A** and B show the results of PCA analysis for serum and fecal samples, respectively. The LC-MS data were further analyzed using OPLS-DA (**Supplementary Figures 2A–H**) to identify differences in serum and fecal metabolites. The metabolite profiles of control and model mice exhibited clear separation, indicating that CS exposure and IAV infection induce sustained changes in metabolites. The R^2^ and Q^2^ values are shown in **Supplementary Figures 3A–H**. A total of 775 serum and 1,495 fecal differential metabolites were identified at across time points. In **Supplementary Table 3**, the Log2FC values, P-values, and predicted variable importance (VIP) values of differential metabolites in serum and feces are listed. **Figure 7C** presents the Kyoto Encyclopedia of Genes and Genomes (KEGG) enrichment analysis results of serum differential metabolites on day 5 post-infection. Highly significant pathways (rich factor ≥ 0.8) include fatty acid biosynthesis, biosynthesis of unsaturated fatty acids, ether lipid metabolism, and others.

Interestingly, the biosynthesis of unsaturated fatty acids pathway (which includes both saturated precursors and unsaturated products) showed significant alterations starting from the third day post-infection and remained suppressed until the fifteenth day post-infection (**Supplementary Figures 4A–D**). Heat-map visualization revealed a progressive intensification of blue tones among metabolites involved in the biosynthesis of unsaturated fatty acids on days 3 and 5 post-infection; by day 15, these signals remained distinctly blue and had not reverted to the baseline color intensity observed in control mice (**Figure 7D**). In contrast to the extensive changes observed in serum, metabolites associated with the biosynthesis of unsaturated fatty acids pathway in the gut showed no pronounced alterations during infection. Heat-map visualizations are presented in **Supplementary Figure 1C**. Meanwhile, KEGG enrichment analysis of differential metabolites at four time points also failed to identify significant changes in the biosynthesis of unsaturated fatty acids pathway (**Supplementary Figures 4E–H**).

Since serum metabolites involved in the biosynthesis of unsaturated fatty acids were consistently downregulated following viral infection, we investigated the correlation between these differentially abundant fatty acids and pulmonary inflammatory markers. The results showed that these metabolites—including both saturated fatty acids (e.g., palmitic acid, stearic acid) and unsaturated fatty acids—were negatively correlated with the mRNA expression levels of pulmonary inflammatory cytokines and BALF-cell counts (**Figure 7E**). Key unsaturated fatty acids exhibiting this negative correlation comprised alpha-linolenic acid (ALA), oleic acid, linoleate, and cis-11,14-eicosadienoic acid.

To establish a direct link between alterations in gut microbiota and changes in circulating metabolites, we conducted a correlation analysis of metabolites involved in the biosynthesis of unsaturated fatty acids in both fecal and serum samples. ALA, eicosapentaenoic acid (EPA), and palmitic acid (PA) exhibited significant positive correlations (**Figure 7F**), while non-significant results presented in other metabolites (**Supplementary Figure 1D**). Further analysis of the correlation between fecal unsaturated fatty acid metabolites and gut microbiota revealed that fecal ALA and PA levels were negatively correlated with *Candidatus Saccharimonas*, whereas EPA exhibited a positive correlation with *Incertae Sedis* (**Figure 7G**). Notably, similar correlations were observed between these three serum metabolites and gut microbiota (**Supplementary Figure 1E**).

### 3.7 Utilizing random forest analysis for the identification of biomarkers

We aim to identify biological indicators that reflect respiratory viral infections in COPD during the acute stage. Our results suggest that the fifth day post-virus challenge represents the peak of pulmonary inflammation in mice. Therefore, we selected the common differential metabolites observed on the first, third, and fifth days after the virus challenge as preliminary screening candidates, totaling 61 metabolites. The differential metabolites at each time point are illustrated in the Venn diagram (**Figure 7H**).

Next, we used random forest analysis to evaluate the effectiveness of these metabolites in distinguishing the virus-infected group from the control group. The study included healthy mice and mice infected on days 1, 3, and 5. The dataset was partitioned into a training set and a test set in a 4:1 ratio. Within the training set, a 5-fold cross-validation procedure was employed to calculate the average false positive rate (FPR) and true positive rate (TPR) across each fold. Subsequently, the area under the receiver operating characteristic (ROC) curve was constructed utilizing these mean FPR and TPR values.

The analysis results are presented in **Table 2**. The area under the curve (AUC) of 20 metabolites exceeded 0.95. Among them, Isovalerylcarnitine (Car(5:0)), 1-Methylnicotinamide, 2-(Aminomethyl)-4-(tert-butyl)-6-(methylsulfonyl)phenol, 5-(4-Methoxyphenyl)pentanoic acid, 4,6-Dichloro-2-cyclopropyl-5-methylpyrimidine, Glabranine, Dephnoretin, Ornithokinin, Ciclesonide, Valerylcarnitine, and Cyclocytidine achieved an AUC of 1, demonstrating excellent diagnostic efficacy. The normalized relative abundance variation of 1-Methylnicotinamide is shown in **Figure 7I**.

**Table 2:** Results of random forest analysis of differential metabolites.

Metabolite	AUC	Accuracy	Recall	Precision	F1-score
Cyclocytidine	1	1	1	1	1
Isovalerylcarnitine (Car(5:0))	1	1	1	1	1
1-Methylnicotinamide	1	1	1	1	1
2-(Aminomethyl)-4-(tert-butyl)-6-(methylsulfonyl)phenol	1	1	1	1	1
5-(4-Methoxyphenyl)pentanoic acid	1	1	1	1	1
4,6-Dichloro-2-cyclopropyl-5-methylpyrimidine	1	1	1	1	1
Glabranine	1	1	1	1	1
Dephnoretin	1	1	1	1	1
Ornithokinin	1	1	1	1	1
Ciclesonide	1	1	1	1	1
Valerylcarnitine	1	1	1	1	1
2-Docosahexaenoyl-1-palmitoyl-sn-glycero-3-phosphoethanolamine	0.984	0.875	0.875	1	0.933
Piplartine	0.984	0.875	0.875	1	0.933
Glycerophosphocholine	0.984	0.875	0.875	1	0.933
5-Nitro-N-(3-pyridinylmethyl)-2-furamide	0.984	0.875	0.875	1	0.933
Stearic acid	0.984	0.875	0.875	0.938	0.891
Arg-Ser-Arg	0.984	0.875	0.875	0.938	0.891
Hygric acid	0.953	0.875	0.875	1	0.933
3-(4-Fluorophenyl)-2-thioxo-2,3-dihydro-4(1H)-quinazolinone	0.953	0.875	0.875	0.938	0.891
Kuwanon C	0.953	0.875	0.875	0.906	0.877
Entacapone 3-.beta.-D-glucuronide	0.938	0.75	0.75	1	0.857
1,5-Naphthalenediamine	0.938	0.75	0.75	0.821	0.708
Pravastatin	0.938	0.875	0.875	0.766	0.817
Laxapur	0.938	0.75	0.75	0.562	0.643
3’-Amino-3’-deoxythymidine	0.938	0.75	0.75	0.75	0.75
TOBRAMYCIN	0.938	0.75	0.75	0.75	0.75
1H-Indazole-3-carboxamide, N-[(1S)-1-(aminocarbonyl)-2-methylpropyl]-1-pentyl-	0.922	0.875	0.875	1	0.933
Cyanazine	0.922	0.875	0.875	1	0.933
2-Arachidonoyl-1-palmitoyl-sn-glycero-3-phosphoethanolamine	0.922	0.875	0.875	0.917	0.882
Tryptophan	0.891	0.875	0.875	0.938	0.891
N,N-Diethyl-3-(4-methyl-5-sulfanyl-4H-1,2,4-triazol-3-yl)benzenesulfonamide	0.875	0.75	0.75	1	0.857
2-amino-3-({[3-(hexadecanoyloxy)-2-(octadecanoyloxy)propoxy](hydroxy)phosphoryl}oxy)propanoic acid	0.875	0.875	0.875	0.938	0.891
Myrtucommulone B	0.859	0.625	0.625	0.683	0.645
SM(d18:1/20:0)	0.859	0.875	0.875	0.893	0.859
(2-aminoethoxy)[2-[docosa-4.7.10.13.16-pentaenoyloxy]-3-(octadecanoyloxy)propoxy]phosphinic acid	0.844	0.875	0.875	0.938	0.891
Isoniazid alpha-ketoglutaric acid	0.828	0.875	0.875	1	0.933
Thymidine	0.828	0.875	0.875	0.896	0.868
4-Chloro-5,7-diphenyl-7H-pyrrolo[2,3-d]pyrimidine	0.828	0.625	0.625	0.536	0.577
2-Hydroxy-6-methylquinoline-3-carbaldehyde	0.812	0.75	0.75	0.85	0.75
Olopatadine	0.812	0.75	0.75	0.75	0.75
17-Acetoxygrindelic acid	0.797	0.625	0.625	0.906	0.686
SM(d16:1/24:1(15Z))	0.797	0.625	0.625	0.604	0.605
L-Fucitol	0.797	0.75	0.75	0.821	0.708
3,5,6,7,8-Pentamethoxy-2-(7-methoxy-1,3-benzodioxol-5-yl)chromen-4-one	0.766	0.625	0.625	0.683	0.645
15-Cyclohexylpentanorprostaglandin F2.alpha.	0.766	0.625	0.625	0.683	0.645
3-(2,3,4-Trimethoxyphenyl)propanoic acid	0.75	0.75	0.75	0.833	0.733
5(S),6(R)-Lipoxin_A4	0.734	0.625	0.625	1	0.769
Phenol, 3-fluoro-4-(trifluoromethoxy)-	0.734	0.625	0.625	0.683	0.645
Benzyl nicotinate	0.719	0.5	0.5	1	0.667
2-(4-(Methylamino)phenyl)benzo[d]thiazol-6-ol	0.719	0.75	0.75	0.75	0.75
cis-11-Eicosenoic acid	0.672	0.5	0.5	0.625	0.533
Protubonine A	0.672	0.625	0.625	1	0.769
1H-Indazole-3-carboxamide, N-[(1S)-1-(aminocarbonyl)-2,2-dimethylpropyl]-1-(5-fluoropentyl)-	0.656	0.5	0.5	0.357	0.417
Glycerophosphoethanolamine	0.641	0.5	0.5	1	0.667
Fulvestrant 9-sulfone	0.625	0.5	0.5	0.5	0.5
Orotic acid	0.609	0.5	0.5	0.625	0.533
[2-[4-Methyl-2-(2-methylpropanoyloxy)phenyl]oxiran-2-yl]methyl 2-methylpropanoate	0.578	0.625	0.625	0.812	0.607
6-Hydroxy-6-(3-hydroxy-2,3-dimethylcyclopentyl)-2-methylheptanoic acid	0.562	0.75	0.75	1	0.857
3,5-Dimethoxy-2,7-phenanthrenediol	0.531	0.5	0.5	0.625	0.533
(3-Ethyl-2-imino-2,3-dihydro-1H-benzimidazol-1-yl)acetic acid	0.516	0.375	0.375	0.141	0.205
Ethynylestradiol 17-.beta.-D-glucuronide	0.5	0.5	0.5	1	0.667

Finally, we conducted the same analysis on the differential flora at the genus level. Unfortunately, no suitable microbial biomarkers for characterizing viral infection-induced AECOPD were identified from the microbiota changes, as none of the bacterial groups exhibited an AUC greater than 0.95.

## 4 Discussion

AECOPD represents a critical event in the natural course of COPD. Virus-associated AECOPD constitutes a significant yet under-researched domain. Gut microbiota and metabolites are increasingly recognized as closely involved in immune responses during influenza virus infection and smoking exposure (Xu et al., 2023; Xing et al., 2025; Zhou et al., 2025). However, to date, no studies have examined the combined effects of cigarette smoke exposure and IAV infection on gut microbiota and metabolite profiles. The present study investigated the temporal patterns and interrelationships among airway immunity, inflammation, structure, and lung function, alongside alterations in gut microbiota and metabolites, in mice exposed to cigarette smoke and infected with IAV from day 1 through day 15 post-infection. Our objectives were to elucidate the characteristics of virus-induced immune-inflammatory responses and gut microbiota–metabolite perturbations under cigarette smoke exposure, and to identify potential characteristic biomarkers throughout the course of combined smoking and influenza virus infection.

In this study, mice exposed to cigarette smoke for two months exhibited significant recruitment of pulmonary immune cells, restricted ventilatory function, and typical emphysematous changes following viral infection, with peak viral load, presenting characteristics resembling AECOPD. Notably, although immune cell counts in BALF and interstitial immune cell levels both peaked on day 5 post-infection, BALF immune cell counts returned to baseline by day 15 (**Figure 2B**). In contrast, pathological inflammation scores and interstitial immune cell counts remained elevated at day 15 post-infection, predominantly consisting of macrophages and lymphocytes (**Figures 3B, E**). This discrepancy reflects a phenotypic transition of immune cell populations between the acute and late inflammatory phases. During the late inflammatory phase, these macrophages may polarize from pro-inflammatory (M1) to anti-inflammatory and pro-resolving (M2) phenotypes. Their persistent presence in the interstitium contributes to tissue remodeling, efferocytosis (the clearance of apoptotic cells), and the repair of inflammation-induced tissue injury (Li et al., 2023). Meanwhile, lymphocytes likely transition from an acute predominance of cytotoxic lymphocytes to effector memory T cells. These cells tend to establish local tissue residency by differentiating into tissue-resident memory T cells (TRM), which persist in the interstitium and serve as sentinels against reinfection by the same pathogen (Lee et al., 2024). Interestingly, mice exhibited a maximum body weight loss of 10% following influenza virus infection, with gradual recovery beginning after day 10 (**Figure 3C**). This weight loss serves as a physiological indicator of systemic inflammatory burden. Notably, this trend mirrored changes in airway lavage fluid cellular profiles, reflecting the fundamental process of immune cell recruitment from systemic circulation into local tissue compartments. Meanwhile, alterations in pulmonary interferons, chemokines, and cytokines appeared to precede immune cell recruitment (**Table 1**), suggesting the classical immunological cascade in which influenza virus-infected epithelial cells upregulate interferon, chemokine, and inflammatory factor expression to recruit immune cells to tissue sites for pathogen clearance.

This study further revealed that influenza virus infection exacerbates the destruction of pulmonary parenchymal architecture, with the onset of such damage coinciding temporally with peak viral load and inflammatory responses. This damage persisted through day 15 post-infection without resolution (**Figure 4C**). These findings suggest that influenza virus infection may constitute a significant contributing factor to emphysema pathogenesis. Consistent with this observation, excessive collagen deposition in tissue emerged between days 5 and 15 (**Figure 4D**). This fibrotic response may represent a transient and dynamic reparative process, reflecting the coexistence of tissue injury and repair, or alternatively, it may constitute a component of airway remodeling in COPD (Shaykhiev et al., 2009; Beers and Morrisey, 2011).

The gut microbiota exerts immunomodulatory functions. In the present study, despite the absence of evident immune cell recruitment or pro-inflammatory cytokine expression at day 1 post-infection, significant alterations were already observed in select gut microbiota, including *Proteobacteria*, *Actinobacteriota*, *Alistipes*, *Ralstonia*, and *Stenotrophomonas* (**Figure 5D**). These findings indicate that, under cigarette smoke exposure, these bacterial taxa exhibit exquisite sensitivity to influenza virus infection and may have potential predictive value for virus-induced AECOPD. Conversely, *Lachnospiraceae_NK4A136* and *Lachnospiraceae_UCG-006* exhibited significant elevation exclusively at day 15, suggesting potential associations with immune resolution and tissue repair functions. LEfSe analysis, employing a multilevel hierarchical framework, further characterized dominant bacterial taxa at distinct temporal stages post-infection, not only at the Phylum and Genus levels.

Notably, a prospective clinical study similarly reported an enrichment of *Alistipes* and *Stenotrophomonas* in AECOPD cohorts (Yan et al., 2024), indicating that these taxa may serve as conserved microbial signatures of COPD exacerbation rather than virus-specific biomarkers. Intriguingly, *Intestinimonas* has been rarely documented in previous COPD investigations. In the present study, the relative abundance of *Intestinimonas* increased significantly on days 3 and 5 post-exacerbation—precisely coinciding with peak pulmonary viral replication and inflammatory responses. Subsequent correlation analyses further demonstrated positive associations between its abundance and multiple inflammatory and immunological parameters. *Intestinimonas* is a Gram-positive, strictly anaerobic bacterium belonging to the phylum Firmicutes, possessing the distinctive capacity to convert dietary fructoselysine into butyrate and acetate (Bui et al., 2015; Rampanelli et al., 2025). Based on these collective findings, we propose that *Intestinimonas* abundance may represent a potential biomarker for predicting the severity of pulmonary inflammation in the context of combined cigarette smoke exposure and influenza virus infection.

Under conditions of cigarette smoke exposure and IAV infection, untargeted metabolomic analysis revealed differential metabolites in serum and fecal samples across four time points. We observed a sustained downregulation of unsaturated fatty acid metabolic pathways in the serum of AECOPD mice from day 3 through day 15 post-exacerbation. Downregulated metabolites included ALA from the ω-3 family, EPA, and docosahexaenoic acid (DHA), as well as linoleic acid and other polyunsaturated fatty acids from the ω-6 family. These findings are consistent with clinical studies reporting decreased linoleic acid levels in AECOPD patients (van der Does et al., 2019; Nambiar et al., 2021; Peng et al., 2023; Shi et al., 2024). Notably, these persistently downregulated serum metabolites remained relatively stable in intestinal contents (**Supplementary Figure 1C**). This discrepancy between intestinal and serum compartments indicates that the observed decline in serum unsaturated fatty acids does not primarily reflect reduced dietary intake but rather diminished systemic bioavailability—potentially due to impaired intestinal absorption, increased peripheral utilization, or a combination of both mechanisms. However, our results revealed positive correlations between serum polyunsaturated fatty acids (PUFAs)—including ALA, EPA, and PA—and their corresponding fecal levels, indicating that serum concentrations of ALA, EPA, and PA are influenced by intestinal levels of these fatty acids. Further analysis of the correlation between fecal unsaturated fatty acid metabolites and gut microbiota showed that fecal ALA and PA levels were negatively correlated with *Candidatus Saccharimonas*, whereas EPA exhibited a positive correlation with *Incertae Sedis*. Moreover, similar correlations were observed between these three serum metabolites and gut microbiota (**Supplementary Figure 1E**). According to the literature, ALA, EPA, and PA are primarily derived from dietary sources, while the gut microbiota mainly influence lipid absorption and metabolism (Semova et al., 2012; Martinez-Guryn et al., 2018; Kumar et al., 2025). Therefore, we propose that *Candidatus Saccharimonas*, *Prevotella*, and *Incertae Sedis* may play a role in maintaining the absorption and metabolism of ALA, EPA, and PA.

Using untargeted serum metabolomics combined with random forest modeling, we identified 20 differential metabolites with an area under the receiver operating characteristic curve exceeding 0.95, enabling potential discrimination of virus-driven acute exacerbations. Among these, 1-Methylnicotinamide (1-MNA), a metabolic product of nicotinic acid (vitamin B3), plays a crucial role in energy metabolism and cell repair (Gebicki et al., 2003). 1-MNA is a downstream product of the interferon-gamma (IFN-γ) signaling pathway. During viral infections, IFN-γ levels are significantly elevated, enhancing the activity of nicotinamide N-methyltransferase (NNMT) and thereby promoting the production of 1-MNA (Gebicki and Wieczorkowska, 2020). 1-MNA exhibits independent anti-inflammatory, antioxidant, and antithrombotic effects and contributes to tissue protection in an IFN-γ-mediated inflammatory environment (Blazejczyk et al., 2016). Importantly, 1-MNA inhibits the activation of NLRP3 inflammasomes in human macrophages (Sidor et al., 2023). These inflammasomes are directly activated by viral proteins in respiratory viral infections, driving the release of IL-1β and IL-18, amplifying the inflammatory cascade, and exacerbating lung injury (Ichinohe et al., 2013).

Interestingly, we observed a significant upregulation of IFN-γ only at the peak of infection (day 3), whereas a marked increase in 1-MNA was detected as early as the initial infection phase (day 1). It is well established that respiratory viral infections induce greater oxidative stress than bacterial respiratory infections. Importantly, substantial evidence suggests that cigarette smoke significantly amplifies oxidative stress responses following viral infection by increasing reactive oxygen species (ROS) production and impairing antioxidant defense mechanisms (Brassington et al., 2022; Wang Y. et al., 2023). It has been reported that oxidative stress influences the AMPK-SIRT1 axis through NAD+ depletion, directly enhancing the expression of NNMT and thereby increasing the concentration of 1-MNA (Yang et al., 2010; Guo et al., 2024). This mechanism explains the observed accumulation of 1-MNA during the first five days of infection: in the early stages of viral infection following cigarette smoke exposure, oxidative stress initially upregulates 1-MNA expression, which is subsequently amplified by a substantial secretion of IFN-γ during the peak of virus-induced inflammation, further promoting the accumulation of this metabolite. Given that 1-MNA functions as a specific anti-inflammatory agent in the context of viral infection and oxidative stress, and that there is currently no direct evidence of 1-MNA upregulation in respiratory bacterial infections, we consider 1-MNA a promising biomarker for AECOPD associated with viral infection warranting further investigation.

One limitation of this study is that only female mice were used as experimental subjects. According to reports by Anthony et al., male and female mice exhibit differences in gut microbiota, and exposure to cigarette smoke induces distinct alterations in these microbial populations (Tam et al., 2020). Furthermore, females demonstrate a stronger immune response to influenza virus than males (Klein et al., 2012). Consequently, the data presented here more accurately reflect the series of immune, microbiota, and metabolite alterations induced by combined smoking and influenza virus infection in females, which may differ from the responses observed in males.

## 5 Conclusions

Mice exposed to cigarette smoke for two months exhibited significant recruitment of pulmonary immune cells between days 3 and 5 post-infection, accompanied by impaired ventilatory function and characteristic features of pulmonary emphysema. The viral load peaked during this period, displaying characteristics similar to AECOPD. By day 15 post-infection, the virus was largely cleared, and acute airway inflammation had mostly subsided; however, chronic pulmonary inflammation persisted, primarily characterized by macrophage infiltration, while signs of pulmonary emphysema remained. Integrated longitudinal multi-omics analysis revealed that gut microbiota and metabolites are potential biomarkers for virus-triggered AECOPD, and supplementation with unsaturated fatty acids may represent a potential therapeutic targets for COPD-related inflammation.

## Data Availability

The original contributions presented in the study are included in the article/**Supplementary Material**. Further inquiries can be directed to the corresponding authors.

## Supplementary material

Supplementary Figure 1 **(A)** Venn diagrams of OTUs clustered in cecal content samples from different groups. **(B)** Rarefaction curves of cecal content samples. **(C)** Heatmap showing differential expression of metabolites in the stool unsaturated fatty acid pathway between the control and model groups. **(D)** Correlation of metabolites involved in the biosynthesis of the unsaturated fatty acid metabolic pathway between feces and serum (negative correlations only). **(E)** Heatmap illustrating the correlation between gut microbiota at the genus level and serum metabolites in the biosynthesis of the unsaturated fatty acid pathway.

Supplementary Figure 2 **(A–D)** OPLS-DA analysis results of serum metabolites. **(E–H)** OPLS-DA analysis results of fecal metabolites.

Supplementary Figure 3 **(A–D)** R^2^ and Q^2^ of serum metabolites OPLS-DA analysis. **(E–H)** R^2^ and Q^2^ of fecal metabolites OPLS-DA analysis.

Supplementary Figure 4 **(A–D)** KEGG enrichment analysis based on differential serum metabolites. **(E–H)** KEGG enrichment analysis based on differential fecal metabolites.

Supplementary Table 1 Scoring criteria for lung tissue inflammation.

Supplementary Table 2 The used primer sequences for RT-PCR in the current study.

Supplementary Table 3 The Log2FC, P-value, and variable importance in projection (VIP) values of the serum and fecal differential metabolites.

## Glossary

1-MNA: 1-Methylnicotinamide
AECOPD: Acute Exacerbations of Chronic Obstructive Pulmonary Disease
ALA: Alpha-linolenic acid
AMPK: AMP-activated protein kinase
ANOVA: Analysis of Variance
AUC: Area Under the Curve
BALF: Bronchoalveolar Lavage Fluid
COPD: Chronic Obstructive Pulmonary Disease
CS: Cigarette Smoke
Ct: Threshold cycle
DAPI: 4’,6-diamidino-2-phenylindole
DHA: Docosahexaenoic acid
EF50: Expiratory Flow at 50% of Vital Capacity
EPA: Eicosapentaenoic acid
ESI: Electrospray Ionization
FPR: False positive rate
H&E: Hematoxylin and Eosin
HRP: Horseradish peroxidase
IAV: Influenza A Virus
KEGG: Kyoto Encyclopedia of Genes and Genomes
LDA: Linear Discriminant Analysis
LEfSe: Linear Discriminant Analysis of Effect Size
Lm: Linear Intercept
MEM: Minimal Essential Eagle’s Medium
MMP-12: Matrix Metalloproteinase-12
NLRP3: NOD-like receptor protein 3
NNMT: Nicotinamide N-methyltransferase
NP: Viral nucleoprotein
OPLS-DA: Orthogonal Projections to Latent Structures Discriminant Analysis
OTUs: Operational Taxonomic Units
PA: Palmitic acid
PBS: Phosphate-buffered saline
PCA: Principal Component Analysis
PCoA: Principal Coordinates Analysis
PEF: Peak Expiratory Flow
Penh: Enhanced Pause
PFU: Plaque-forming units
PUFAs: Polyunsaturated fatty acids
QC: Quality control
RCF: Relative Centrifugal Force
ROC: Receiver operating characteristic
ROI: Region of interest
ROS: Reactive oxygen species
RT-PCR: Real-time quantitative PCR
SEM: Standard Error of the Mean
SIRT1: Sirtuin 1
SNCE: Stepwise Nanospray Collision Energy
SPF: Specific pathogen-free
TE: Expiratory Time
TPR: True positive rate
TRM: Tissue-resident memory T cells
Tr: Relaxation Time
TSA: Tyramide signal amplification
TV: Tidal Volume
UHPLC: Ultra-High Performance Liquid Chromatography
VIP: Variable importance in projection.
