# Supplementary figures and images for: Longitudinal multi-omics evidence reveals lung injury and concurrent disruption of intestinal flora and serum metabolism by cigarette smoke and influenza virus
Source: Front Cell Infect Microbiol. 2026 Apr 7;16:1731390. doi: 10.3389/fcimb.2026.1731390 (PMC13095677; doi:10.3389/fcimb.2026.1731390)

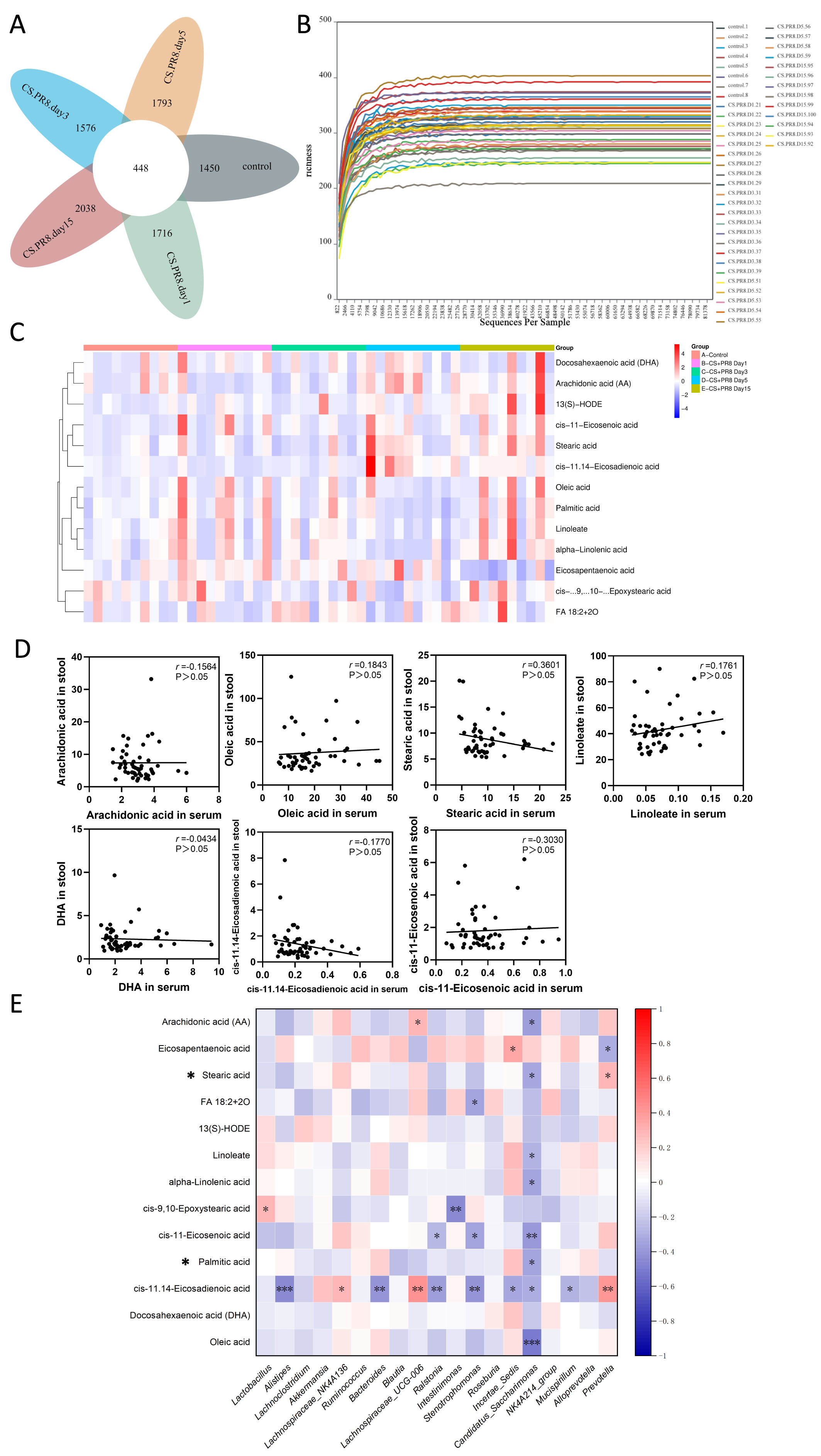

Supplement: Supplementary Figure 1 — (A) Venn diagrams of OTUs clustered in cecal content samples from different groups. (B) Rarefaction curves of cecal content samples. (C) Heatmap showing differential expression of metabolites in the stool unsaturated fatty acid pathway between the control and model groups. (D) Correlation of metabolites involved in the biosynthesis of the unsaturated fatty acid metabolic pathway between feces and serum (negative correlations only). (E) Heatmap illustrating the correlation between gut microbiota at the genus level and serum metabolites in the biosynthesis of the unsaturated fatty acid pathway. [file Image1.tif]

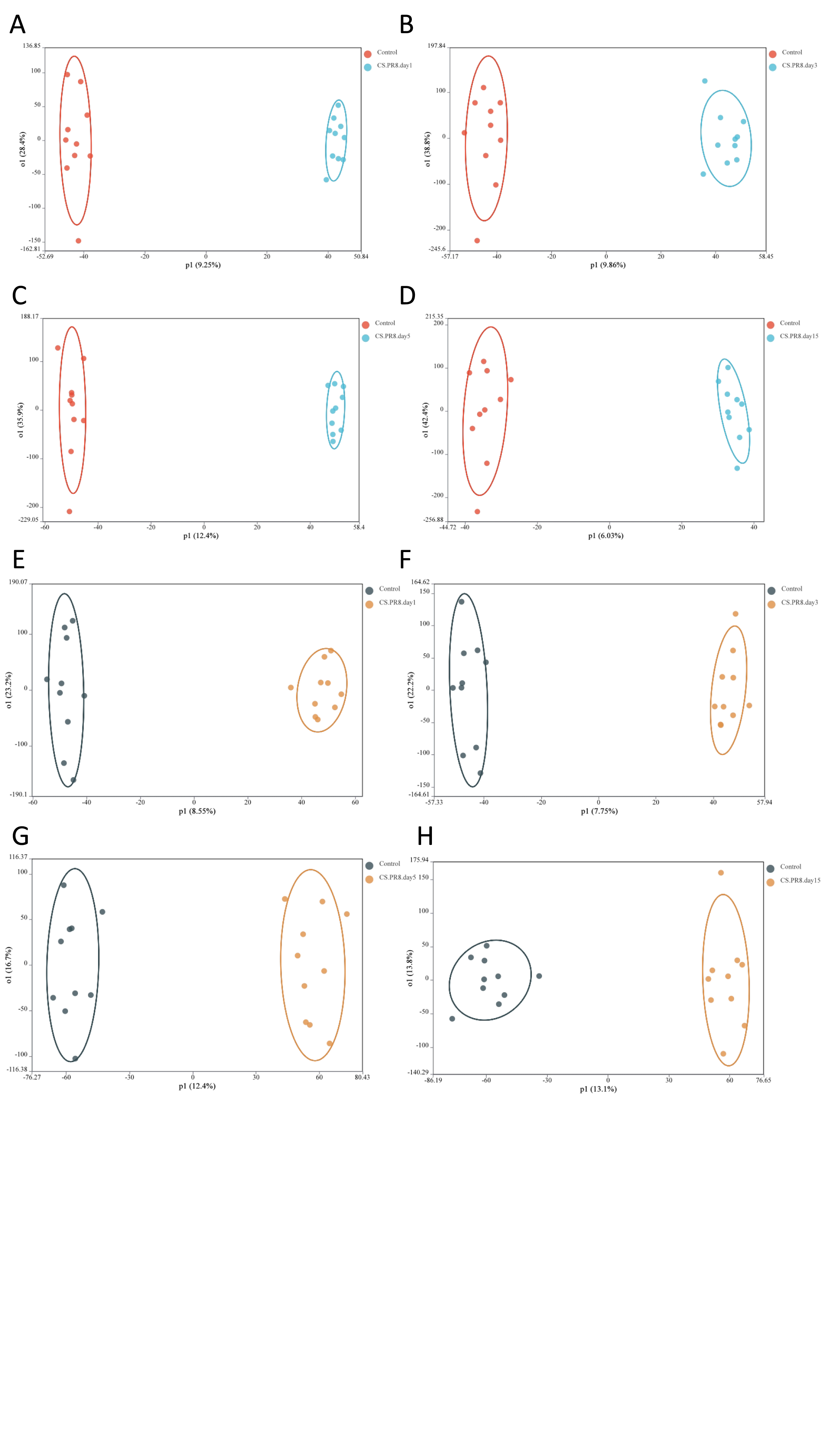

Supplement: Supplementary Figure 2 — (A–D) OPLS-DA analysis results of serum metabolites. (E–H) OPLS-DA analysis results of fecal metabolites. [file Image2.tif]

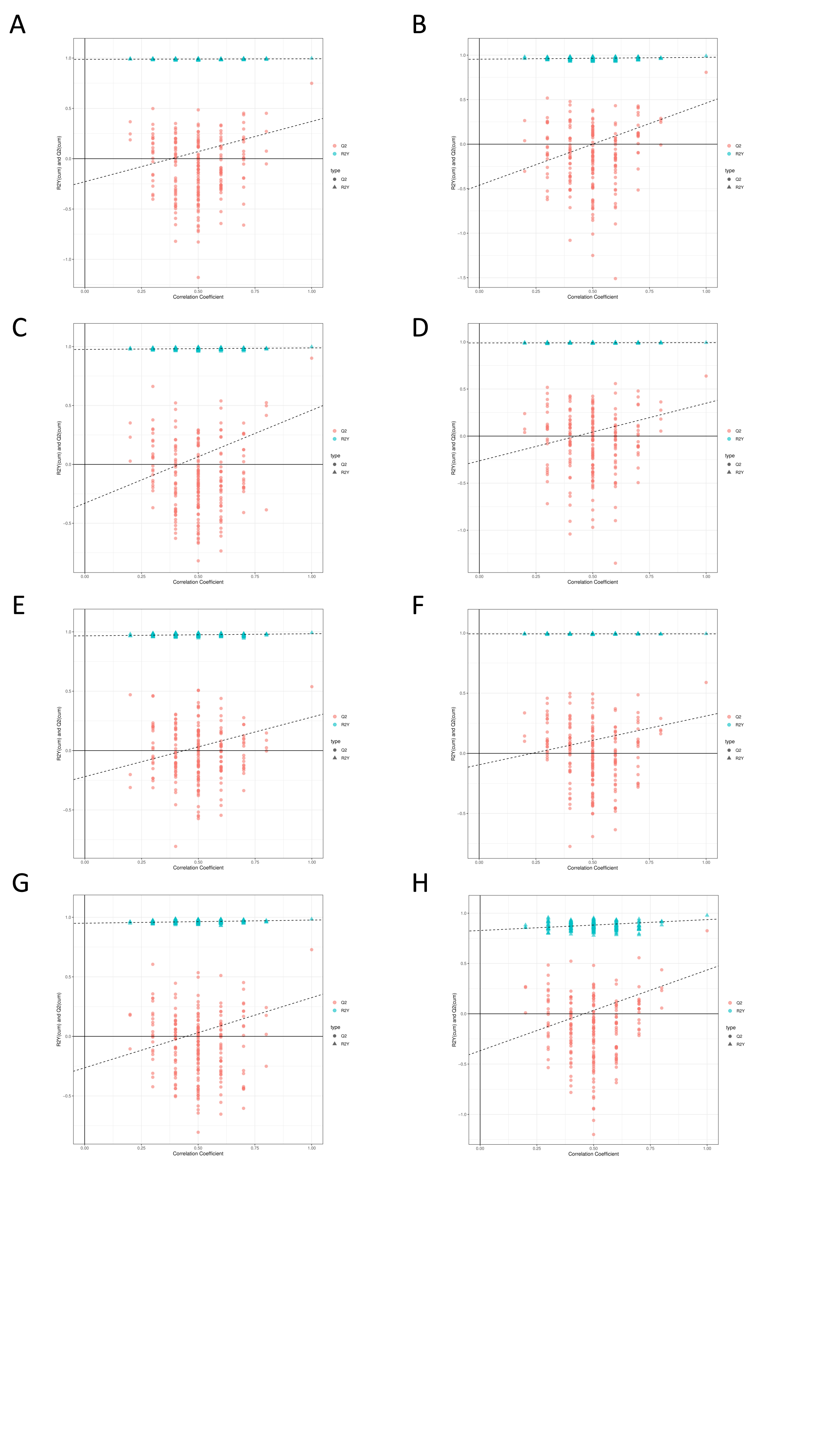

Supplement: Supplementary Figure 3 — (A–D) R2 and Q2 of serum metabolites OPLS-DA analysis. (E–H) R2 and Q2 of fecal metabolites OPLS-DA analysis. [file Image3.tif]

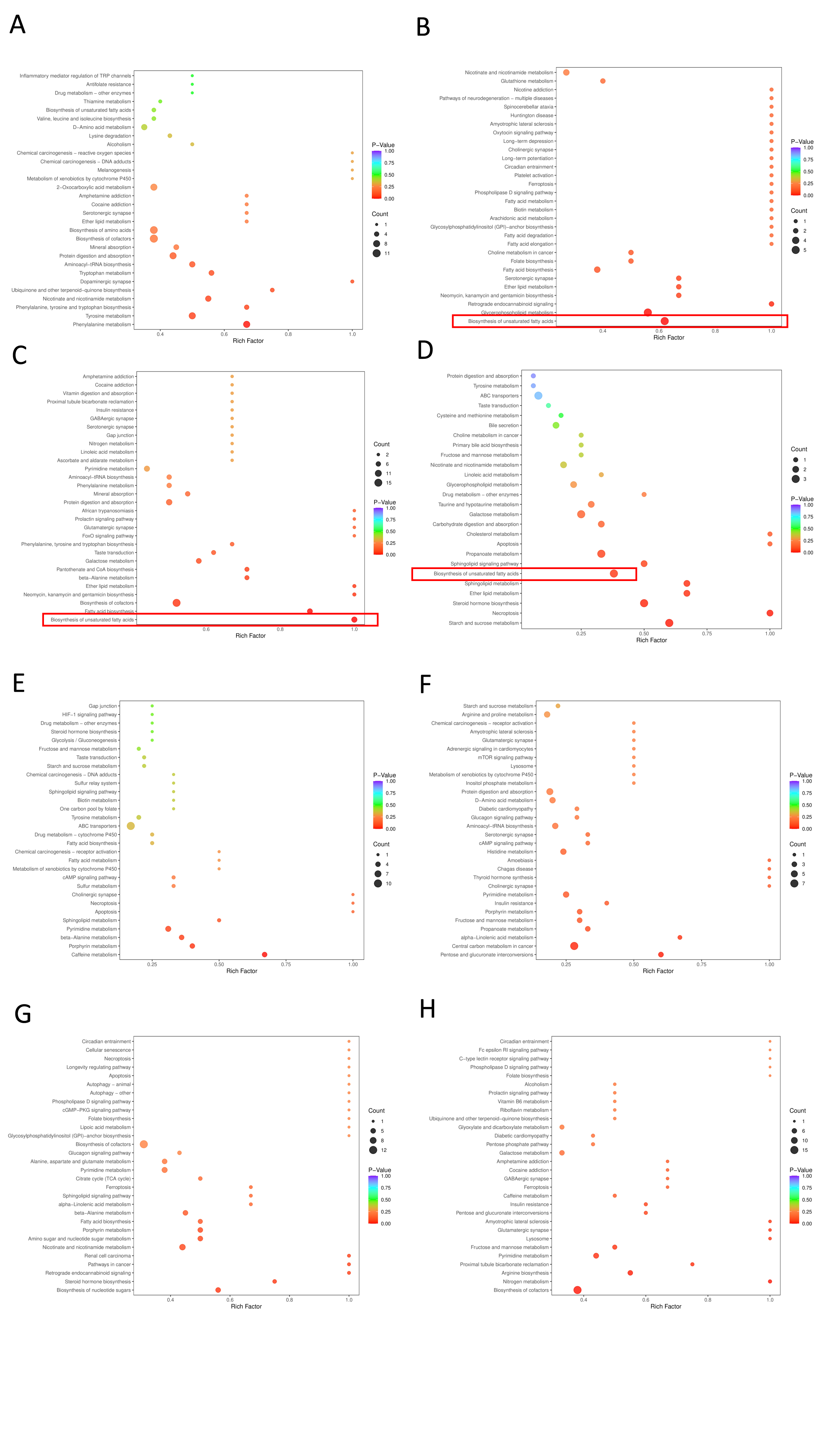

Supplement: Supplementary Figure 4 — (A–D) KEGG enrichment analysis based on differential serum metabolites. (E–H) KEGG enrichment analysis based on differential fecal metabolites. [file Image4.tif]
